# Laparoscopic ovariectomy in dogs in late gestation

**DOI:** 10.1186/s12917-018-1770-z

**Published:** 2019-01-08

**Authors:** Kaustubh R. Dongaonkar, Sarita U. Gulavane, Veeraraghavan M. Chariar, Kiran R. Shelar

**Affiliations:** 1 Department of Animal Reproduction, Gynaecology & Obstetrics, Bombay Veterinary College, Maharashtra Animal and Fishery Sciences University, Mumbai, Maharashtra 400012 India; 2 Dr Chariar’s Pet Specialty Clinic, Thane, Maharashtra 400607 India; 3 Present Address: Department of Veterinary Clinical Sciences, School of Veterinary Medicine, Louisiana State University, Skip Bertman Drive, Baton Rouge, LA 70803 USA

**Keywords:** Laparoscopic ovariectomy, Pregnant dogs, Stray dog sterilization

## Abstract

**Background:**

Surgical sterilization of stray dogs is the most widely used technique to control stray dog population. Although ovariectomy is an effective technique for elective sterilization of female dogs, most stray dog population control programs generally utilize ovariohysterectomy for spaying female dogs. In the context of stray dog sterilization, laparoscopic ovariectomy has been utilized and reported to be cost-effective compared to programs utilizing open surgical approaches. However, when pregnant stray dogs are encountered either conventional ovariohysterectomy is performed or surgery is deferred altogether. It is reported that ovariectomy at any stage during canine pregnancy results in fetal resorption or abortion, however, clinical outcomes following laparoscopic ovariectomy in dogs in late gestation have not been previously reported. The purpose of this study was to investigate the outcome of laparoscopic ovariectomy with intra-gestational sac injection (IGSI) of potassium chloride (KCl) in dogs in late gestation.

**Results:**

Eight client owned dogs in the gestational age range of 40–50 days underwent laparoscopic ovariectomy with IGSI of KCl. Laparoscopic ovariectomy resulted in decreased serum progesterone from 11.6 ± 2.6 ng/ml on day 0 to 1.3 ± 0.4 ng/ml 24 h’ post-surgery. IGSI of KCl did not result in immediate fetal death and fetal death temporally closely followed the drop in serum progesterone noted 24 h post-ovariectomy. Viscous brown vulvar discharge preceded fetal expulsion by 12 h and all the fetuses were dead when expelled. Uterine evacuation was documented within 45 ± 20 h (1–3.5 days) in all dogs without any clinically significant complications.

**Conclusion:**

Laparoscopic ovariectomy enables elective termination of pregnancy and simultaneous neutering of dogs in late gestation and has potential applications in high volume stray dog sterilization programs which utilize laparoscopy. Utility of IGSI of KCl in this regard is unclear.

## Background

Surgical sterilization of stray dogs is the most widely used technique to control stray dog population [1]. Free-ranging dogs and rabies transmission are integrally linked, and large, unmanaged dog populations can be daunting to rabies control programs [1]. Dog population control programs generally utilize ventral midline or flank approaches for performing ovariohysterectomy. Duration of hospitalization for stray dogs following conventional ovariohysterectomy ranges from 3 to 10 days [2–4] and tends to be significantly longer compared to pet dogs due to concerns following release for self-mutilation, wound dehiscence, myiasis, lack of supervision and lack of follow-up. It is reported that stray dogs spend an average of 12.7 days in the facility from capture to release, which incurs higher running costs and limits the number of surgeries that can be performed during a calendar year in the available kennel space [5]. In addition, pregnant dogs are frequently encountered [6, 7] and undergo ovariohysterectomy in most stray dog sterilization centres. Incisional length and duration of hospitalization both tend to be generally longer in the population of pregnant stray dogs that undergo ovariohysterectomy [3].

Ovariectomy is an effective technique for elective sterilization of female dogs with no recognized disadvantages [8]. In small animal practice, laparoscopic ovariectomy is a commonly performed procedure, and is associated with a shorter recovery time than open ovariectomy [9]. In the context of stray dog sterilization, it is reported that laparoscopic ovariectomy reduces the average duration of hospitalization to 3 days/female dog from capture to release from the surgical facility [5, 10]. Despite the initial costs of equipment, the high volume stray dog laparoscopic spay program compares favourably to programs utilizing conventional ovariohysterectomy by enabling more surgeries to be performed during a calendar year in the available kennel space and a comparatively lower cost/dog spayed [5].

Ovariectomy at any stage during canine pregnancy results in fetal resorption or abortion [11]. It is reported that ovariectomy at ≥ day 32 of pregnancy results in abortion within 2–8 days post-operatively [11] and the interval between ovariectomy and abortion shortens with advancing gestation [12]. Laparoscopic ovariectomy when performed in dogs < 30 days of gestation results in fetal resorption in 3–12 days and is reported to be a safe procedure for neutering such dogs with potential applications in high volume laparoscopic spay programs [13, 14]. To authors’ knowledge, there are no reports on laparoscopic ovariectomy in dogs in late gestation.

Transabdominal ultrasound guided fetal intra-cardiac potassium chloride (KCl) injection for twin reduction has been reported in mares [15, 16]. To authors’ knowledge there is no data available on induction of fetal demise prior to abortion in dogs.

The objective of this study was to describe and evaluate a technique for laparoscopic ovariectomy with intra-gestational sac injection (IGSI) of KCl in pregnant dogs in the gestational age range of 40–50 days. We hypothesised that laparoscopic ovariectomy with IGSI of KCl in dogs in late gestation would result in termination of pregnancy and enable simultaneous neutering. We further hypothesised that IGSI of KCl will induce immediate fetal death.

## Methods

This study was approved by the Board of Studies, Maharashtra Animal & Fishery Sciences University, India. This report is a case series of eight mixed-breed, young to middle-aged, shelter adopted, client owned, pregnant dogs weighing 20 to 30 kg, and in the gestational age range of 40–50 days that underwent laparoscopic ovariectomy and IGSI of KCl. A written informed consent was obtained for each dog from each owner. Dogs were hospitalized one day prior to surgery and monitored for 7 days post-operatively or until documentation of uterine evacuation. While in hospital dogs were housed individually in kennels of appropriate size, walked outside on a leash q8 hours, provided with free choice water and fed commercial canine adult maintenance diet. Following documentation of uterine evacuation all dogs were discharged from the hospital to return to their respective owners and homes.

### Ultrasound

Pregnancy was confirmed by ultrasound (Aloka SSD 500/3.5 MHz sector probe). Fetal bi-parietal diameter (BPD) was measured using electronic callipers and readings were recorded for a minimum of two fetuses for each dog; average fetal BPD for each dog was recorded. Gestational age (GA) was determined using the formula: GA = (BPD × 15) + 20 [17]. Post-operatively, dogs were hospitalized for up to a week and abdominal ultrasound was performed q12 h for each patient for documentation of fetal death and until documentation of complete uterine evacuation. Fetal death was determined to occur when no fetal cardiac activity was observed on ultrasound. Uterine evacuation was determined to occur when no fetus or uterine contents were observed on ultrasound.

### Anesthesia

All dogs received a complete general physical examination and had a complete blood cell count and biochemistry profile evaluated. Food was withheld from each dog for a minimum of 12 h prior to induction of anesthesia. Dogs were premedicated with acepromazine (0.1 mg/kg SC) and atropine (0.04 mg/kg SC). General anesthesia was induced with 2.5% thiopentone sodium (10–25 mg/kg IV to effect) and maintained using 2.5% thiopentone sodium to effect. Endotracheal intubation was performed and intermittent positive pressure ventilation was provided using a bag valve mask and supplemental oxygen. Lumbosacral epidural analgesia was administered using aseptic technique with 4 mg/kg of 2% lidocaine and 2 mg/kg ketamine [18]. Lactated Ringer’s solution was given intravenously for the duration of surgery (10 mL/kg/hr). Heart rate, respiratory rate, temperature, indirect blood pressure, haemoglobin saturation, and electrocardiography were monitored during anesthesia. Postoperative pain management was provided using tramadol (2–3 mg/kg SC or PO q8–12 hours/ PRN).

### Surgery

Dogs were placed in dorsal recumbency in reverse Trendelenburg position on a custom-made positioner. The ventral abdomen was prepared and draped for aseptic surgery. A 5 mm trocar was inserted directly at the midline just caudal to the umbilicus by elevating the rectus sheath [19]. Adequate capno-peritoneum was created and maintained under 12–15 mmHg pressure using a mechanical insufflator (CO_2_ Endoflator SCB - Karl Storz Veterinary Endoscopy, Goleta, CA). The umbilical port housed a 5 mm Hopkins II forward-oblique telescope 30° (Karl Storz Veterinary Endoscopy) connected to an endoscopic camera (Veterinary Video Camera III - Karl Storz Veterinary Endoscopy) and light source (Xenon nova - Karl Storz Veterinary Endoscopy). Two additional midline ports, one 4–5 cm cranial and another 4–5 cm caudal to the first port were placed under laparoscopic guidance through which 5 mm curved Maryland dissecting forceps and 5 mm toothed grasping forceps were introduced respectively. The dissecting forceps were connected to an electrosurgical unit, (Autocon 200 - Karl Storz Veterinary Endoscopy). Laparoscopic ovariectomy was carried out by open bursal technique [20]. For ablation of the left ovary, the dog was positioned in right oblique recumbency. The ovarian parenchyma was exteriorized from the ovarian bursa. Dissection of the ovarian parenchyma was then performed with dissecting forceps using monopolar electro-surgery. The dissected ovary was removed through the most caudal port. The procedure was repeated for the right ovary after rotating the patient to left oblique recumbency. Bilateral laparoscopic ovariectomy was followed by intra-gestational sac injection. The abdominal incisions were closed using 2–0 polydioxanone in simple interrupted pattern on the external rectus sheath, 3–0 polyglactin 910 in a simple interrupted pattern in the subcutaneous and intradermal layer. Cyanoacrylate glue was used on the incision topically.

### Intra-gestational sac injection

A solution of KCl with 1 MEq/ml concentration was made by diluting the commercially available KCl (150 mg/ml) with sterile water. After examination of the entire uterus and ascertaining the number of gestational sacs present, intra-gestational sac injections were performed using a 20 G 1.5 in. needle introduced percutaneously under laparoscopic guidance. Each gestational sac was injected with 1 ml (1MEq) of KCl solution.

### Clinical monitoring

Dogs were monitored for 7 days following surgery or until documentation of uterine evacuation. Monitoring parameters were recorded twice a day and included heart rate, respiratory rate, rectal temperature, mucous membrane color, capillary refill time, mentation, pain score, appetite, urination, nature of feces, presence of vomiting, presence and nature of vaginal discharge and fetal expulsion. Complete blood cell count and biochemistry profile was repeated one week postoperatively.

### Serum progesterone monitoring

Serum progesterone was measured for each patient preoperatively on the day of the surgery (day 0) and on days 1, 3 and 7. The samples were analysed by Fully Automated Bi-Directional Inter-Faced Chemi Luminescent Immuno Assay method (analytical sensitivity ≥0.1 ng/ml).

### Statistical analysis

Data were summarized as mean and standard deviation (SD) for continuous measures.

## Results

### Surgical findings

Laparoscopic ovariectomy was successfully performed in all 8 pregnant dogs without need for conversion to laparotomy. Manipulation of distended uterine horns to access ovarian bursa was noted to be challenging. Patient positioning in a laterally oblique recumbency with the aid of a positioner and reverse Trendelenburg position provided the optimal operating space. None of the dogs had any evidence of complications following port placement. Laparoscopic examination of 40–50 days’ pregnant dogs revealed moderately distended uterine horns containing poorly demarcated oblong and fluctuant gestational sacs with central dark transverse bands indicating the sites of zonary placentation. None of the dogs needed any additional measures for hemostasis for performing laparoscopic ovariectomy. Litter size ranged from 4 to 8 and accordingly the total dose of KCl administered via IGSI ranged from 4 to 8 ml/dog (4–8 MEq/dog). IGSI of KCl was administered without any complications for the dogs.

### Ultrasound findings

Estimated gestational age based on pre-operative ultrasound scans ranged from 41 days to 48 days. On the 12-h post-operative scans, 35/42 (83%) fetuses were noted to be alive with fetal heart rates within normal reference range. At 24-h post-operative scans, 37/42 (88%) fetuses were noted to be dead; and 2/8 dogs were noted to have undergone complete uterine evacuation (Table 1). Interval between laparoscopic ovariectomy with IGSI of KCl and complete uterine evacuation ranged from 24 to 84 h (1 to 3.5 days) with a Mean ± SD of 45.0 ± 20 h post-surgery.

**Table 1:** Post-operative ultrasound monitoring

Dog	Estimated litter size	No of dead fetuses at 12 h scan	No of dead fetuses at 24 h scan	Uterine evacuation (UE) (hours)
1	5	1/5	3/5	48
2	4	0/4	4/4	36
3	4	0/4	3/4	48
4	4	0/4	4/4	36
5	8	2/8	7/8	60
6	6	0/6	5/6	84
7	6	0/6	UE	24
8	5	4/5	UE	24
				Mean ± SD 45.00 ± 20.03
				Range 1–3.5 days

### Clinical monitoring

All dogs recovered uneventfully from the anesthesia. None of the dogs needed additional rescue analgesia in the post-operative period. Expulsion of dead fetuses along with fetal membranes was noted to occur in all dogs. Viscous brown vulvar discharge preceded fetal expulsion by about 12 h in all dogs. Interval between laparoscopic ovariectomy with IGSI of KCl and expulsion of first dead fetus ranged from 12 to 48 h after surgery. Vulvar discharge continued for 1–3 days after uterine evacuation. Five dogs exhibited transient anorexia and all dogs had signs of mild to moderate mammary congestion. No other complications were encountered during the immediate post-operative period of 7 days and the surgical incisions healed uneventfully in all dogs. The changes in the monitored parameters were unremarkable. The complete blood cell count and biochemical profile were within normal reference range in all dogs both pre and post-operatively.

### Serum progesterone

Mean serum progesterone of dogs in the gestational age range of 40–50 days was 11.62 ± 2.6 ng/ml which dropped to a mean serum progesterone of 1.31 ± 0.4 ng/ml, 0.54 ± 0.2 ng/ml and 0.33 ± 0.2 ng/ml on days 1, 3 and 7 post-ovariectomy respectively. (Table 2).

**Table 2:** Progesterone levels following laparoscopic ovariectomy and IGSI of KCl

Dog	Progesterone ng/ml			
	Day 0	Day 1	Day 3	Day 7
1	14.11	1.25	0.51	0.43
2	11.34	0.96	0.23	0.13
3	13.97	1.95	0.84	0.54
4	6.46	2.14	0.78	0.74
5	13.96	1.15	0.80	0.14
6	11.19	0.96	0.50	0.31
7	9.63	1.15	0.35	0.29
8	12.30	0.92	0.31	0.12
Mean ± SD	11.62 ± 2.63	1.31 ± 0.47	0.54 ± 0.24	0.33 ± 0.22

## Discussion

Clinical outcomes following laparoscopic ovariectomy in dogs in late gestation have not been previously evaluated. In the present study we documented that laparoscopic ovariectomy was technically feasible in dogs in late gestation and enables elective termination of pregnancy and simultaneous neutering.

In dogs, the ovaries are essential throughout pregnancy and plasma progesterone concentration must be ≥2 ng/ml to maintain pregnancy [21]. In the present study, serum progesterone 24 h post-ovariectomy was < 2 ng/ml in all dogs except one in which it was 2.14 ng/ml. This marked decrease in serum progesterone was closely followed by fetal expulsion and complete uterine evacuation in all dogs.

The technique of IGSI of KCl was chosen to induce immediate fetal demise prior to their expulsion. Although, ultrasound guided fetal intra-cardiac KCl has been reported in mares to induce fetal demise [15, 16], this was not attempted in the present study and intra-gestational sac injections were administered with laparoscopic guidance. It is reported that IGSI with normal saline has no adverse effect on canine pregnancy [22] which suggests that IGSI by itself is inconsequential. Contrary to our hypothesis, the results of the present study suggest that IGSI of KCl did not have an immediate detrimental effect on the fetuses or induce fetal demise. Fetal death temporally closely followed the marked decrease in serum progesterone noted 24 h post-ovariectomy. Thus, the need and utility of IGSI is unclear.

The present study did not have a control group with dogs undergoing laparoscopic ovariectomy without IGSI of KCl, and is a limitation of the study. However, time interval between laparoscopic ovariectomy and documentation of uterine evacuation observed in the present study was comparable to previously reported time interval between ovariectomy and abortion for dogs in similar gestational age [11, 12]. Thus, we speculate that IGSI of KCl was inconsequential.

During ovariohysterectomy in pregnant dogs, it is recommended to retain fetuses in the closed uterus for 1 h or longer after removal of the uterus from the dam to prevent fetal suffering. [23–25] However to the authors’ knowledge no guidelines exist for induction of fetal death prior medical termination of pregnancy in dogs and may warrant further research. Several drug protocol have been evaluated for termination of pregnancy in mid-late gestation in dogs (≥40 days) all eventually leading to withdrawal of the activity of progesterone resulting in fetal death, followed by abortion [26–28]. Medical protocols recommended for use in dogs in mid-late gestation involve 3–10 days of daily treatment and commonly result in termination of pregnancy, like in the present study, between 40 and 50 days of gestation [26–28]. Laparoscopic ovariectomy in dogs during late gestation is similar to the medical protocols for termination of pregnancy as it resulted in a precipitous drop in serum progesterone and eventually fetal death and expulsion. Similarly, signs noted during uterine evacuation following laparoscopic ovariectomy such as vulvar discharge, transient anorexia and mammary congestion were consistent with signs reported for medical termination of pregnancy in dogs [26, 28]. In the present study, 88% fetuses were noted to be dead 24 h following laparoscopic ovariectomy and prior to expulsion; all fetuses were noted to be dead at expulsion and complete uterine evacuation occurred between 1 and 3.5 days. Abdominal ultrasound performed at more frequent intervals may have facilitated a more accurate assessment of incidence of fetal death prior to uterine evacuation. Determining the gestational age using ultrasound allowed for careful case selection for performing laparoscopic ovariectomy in dogs in late gestation.

Diagnostic laparoscopy has been reported in dogs during pregnancy with no associated complications or consequences on the viability of the fetuses [29]. Similarly, no anesthetic or procedural complications were encountered in previous studies evaluating laparoscopic ovariectomy in ≤30 days’ pregnant dogs [13, 14]. We acknowledge that the anesthesia and equipment choices in this study were made to replicate resources available at the high volume stray dog laparoscopic spay facility and although not ideal they were, in our experience, effective and safe.

Undertaking surgical sterilization of stray dogs on a large scale is logistically demanding, and in low resource settings where the need to control stray dog populations is often the greatest, it is particularly challenging. A high volume stray dog laparoscopic spay program has been operational in Thane, India since 2004. Despite the initial cost of equipment, data from this centre reveals that a stray dog sterilization program utilizing laparoscopic ovariectomy compares favourably to programs utilizing conventional ovariohysterectomy. The primary advantage of such laparoscopic spay programs is the ability to release stray dogs 24 h following surgery, which in turn allows for more surgeries to be performed during a calendar year with the available kennel space and keeping the costs of the operation/dog spayed to a minimum [5, 10, 13]. The technique reported in the present study primarily has applications in high volume stray dog laparoscopic spay programs and may help facilitate shorter duration of hospitalization of pregnant dogs encountered at such high volume laparoscopic spay centres.

Concerns following medical termination of pregnancy protocols, apart from drug related side-effects, are treatment failure resulting in persistence of luteal tissue and maintenance of adequate progesterone levels resulting in partial abortion and fetal retention [26, 28]. Laparoscopic ovariectomy enables removal of both ovaries and thus ensures complete withdrawal of progesterone function and uterine evacuation. Although lack of long term follow-up is a limitation of the present study, uterine evacuation was documented in 1–3.5 days in all dogs following laparoscopic ovariectomy. We speculate long term complications following documentation of uterine evacuation secondary to laparoscopic ovariectomy in late gestation would be similar to those reported for ovariectomy or ovariohysterectomy in non-pregnant dogs [8].

Limitations of this study, in addition to those previously discussed, include small patient population, lack of long term follow-up, lack of cohort with dogs undergoing laparoscopic ovariectomy without IGSI KCl and lack of documentation of surgery times.

The preferred surgical treatment for pregnancy termination in the dog is ovariohysterectomy. Clinicians are advised to weigh the goals of the owners, available alternatives, signalment and clinical status of the patient, gestational age, and training and experience in minimally invasive surgery when performing laparoscopic ovariectomy in pregnant dogs.

## Conclusions

Laparoscopic ovariectomy enables elective termination of pregnancy and simultaneous neutering of dogs in late gestation and has potential applications in high volume stray dog laparoscopic spay programs. Utility of IGSI of KCl in this regard is unclear.

## Abbreviations

IGSI: Intra-gestational sac injection
KCl: Potassium chloride
SD: Standard deviation
UE: Uterine evacuation
